# Targeting intra-viral conserved nucleocapsid (N) proteins as novel vaccines against SARS-CoVs

**DOI:** 10.1042/BSR20211491

**Published:** 2021-09-24

**Authors:** Min Thura, Joel Xuan En Sng, Koon Hwee Ang, Jie Li, Abhishek Gupta, Jimmy Ming Hong, Cheng William Hong, Qi Zeng

**Affiliations:** 1 Institute of Molecular and Cell Biology, Agency for Science, Technology and Research, Singapore 138673; 2 Lee Kong Chian School of Medicine, Singapore 308232; 3 Department of Radiology, University of California San Diego, San Diego, CA 92103, USA; 4 Department of Biochemistry, Yong Loo Lin School of Medicine, National University of Singapore, Singapore 119260; 5 INTRA-ImmuSG Private Limited, Singapore 079903

**Keywords:** Coronavirus, Nucleocapsid protein, Vaccine

## Abstract

Severe acute respiratory syndrome coronavirus 2 (SARS-CoV-2) has caused the global pandemic of the Coronavirus disease in late 2019 (COVID-19). Vaccine development efforts have predominantly been aimed at 'Extra-viral' *Spike* (*S*) protein as vaccine vehicles, but there are concerns regarding ‘viral immune escape’ since multiple mutations may enable the mutated virus strains to escape from immunity against S protein. The ‘Intra-viral’ Nucleocapsid (N-protein) is relatively conserved among mutant strains of coronaviruses during spread and evolution. Herein, we demonstrate novel vaccine candidates against SARS-CoV-2 by using the whole conserved N-protein or its fragment/peptides. Using ELISA assay, we showed that high titers of specific anti-N antibodies (IgG, IgG1, IgG2a, IgM) were maintained for a reasonably long duration (> 5 months), suggesting that N-protein is an excellent immunogen to stimulate host immune system and robust B-cell activation. We synthesized three peptides located at the conserved regions of N-protein among CoVs. One peptide showed as a good immunogen for vaccination as well. Cytokine arrays on post-vaccination mouse sera showed progressive up-regulation of various cytokines such as IFN-γ and CCL5, suggesting that T_H_1 associated responses are also stimulated. Furthermore, vaccinated mice exhibited an elevated memory T cells population. Here, we propose an unconventional vaccine strategy targeting the conserved N-protein as an alternative vaccine target for coronaviruses. Moreover, we generated a mouse monoclonal antibody specifically against an epitope shared between SARS-CoV and SARS-CoV-2, and we are currently developing the First-in-Class humanized anti-N-protein antibody to potentially treat patients infected by various CoVs in the future.

## Introduction

Coronaviruses are enveloped, positive-sense, single-stranded RNA viruses of the family *Coronaviridae*. While most viruses show mild illnesses such as the common cold, some viruses cause more severe illness, such as SARS-CoV-1 which resulted severe acute respiratory syndrome (SARS) public health crises in 2003, MERS-CoV caused Middle East respiratory syndrome (MERS) in 2009 and SARS-CoV-2 has caused the Coronavirus disease in late 2019 (COVID-19). The outbreaks for SARS-CoV-1 and MERS-CoV were regional, while that of SARS-CoV-2 is global. The World Health Organization (WHO) declared COVID-19 as a pandemic on March 11, 2020 and SARS-CoV-2 has infected more than 177 million people and caused over 3.8 million deaths worldwide as of June 16, 2021 with severe outbreaks occurring first in China, then Europe, USA, South America and Asia [1]. While infections are generally self-resolving in healthy individuals, it can also lead to severe pneumonia, multi-organ failure, and death in significant proportion of infected patients, especially those with pre-existing comorbidities. Along with drastic social distancing measures to slow the spread of the virus, the current COVID-19 pandemic has caused widespread medical, social, political, and financial repercussions.

Furthermore, multiple mutations and variants of SARS-CoV-2 strain have been reported since late 2020 and there are concerns regarding ‘viral immune escape’. It is important for researchers to stay ahead of the pace of viral mutation and another spread. There is an urgency to develop new vaccines against coronaviruses. A vaccine aims to activate acquired immunity to fight against specific pathogens like viruses or bacteria. To resemble a disease-causing microorganism, a vaccine typically is an inactive microbe, or one of its surface proteins to stimulate the body's immune system, which can then remember and recognize ‘foreign antigens’, thus destroying the microorganisms that it may encounter and allowing a stronger response to the active pathogen in the future. Currently, there are several vaccines against COVID-19 mainly mRNA vaccines, viral vector vaccines and conventional inactivated vaccines of which the mRNA and viral vector vaccines target on Spike (S) protein [2], a surface protein that possesses highly evolving glycans [3], which is mostly the key target of vaccine production. However, mutations on S-protein have rendered the development of such conventional vaccine challenging over the past decades. Both Moderna’s mRNA-1273 vaccine and Pfizer-BioNTech’s mRNA vaccine encode for a pre-fusion stabilized form of the S protein. However, the key concern is the efficacy/accuracy of the *Spike mRNAs* being properly translated and glycosylated *in vivo* to mimic its natural Spike protein (an ‘Extra-viral protein’) to trigger host immune systems, producing specific anti-Spike antibodies for viral immunity although these vaccines have been administered in humans across multiple countries worldwide.

Herein, we explore the use of the ‘Intra-viral’ Nucleocapsid (N) protein as a novel vaccine strategy. The N-protein sequence is relatively conserved within any given strain of virus during spread and evolution and across different strains of coronaviruses. The N-protein is functioning as a RNA chaperone and is important for viral transcription, assembly [4] and has high homology with other pathogenic coronavirus family [5]. It is hypothesized that a successful N-protein vaccine will be useful against several viruses within the coronavirus family. However, using these unconventional vaccines targeting ‘Intra-viral’ N-protein may raise questions whether host immune systems are able to detect proteins localized inside viruses to produce specific antibodies. Interestingly, anti-N antibodies were detected in patients’ sera who were diagnosed with SARS-CoV [6] and SARS-CoV-2 infection [7]. This indicates that the N-protein is exposed to be detected by the patients’ immune system in diseased inflammatory states. While the exact mechanism remains to be elucidated, it is possible that infected cells are more fragile and lysed to release the immunogenic N-proteins, and incompletely assembled viral particles may expose the N-protein to the ‘Extra-viral’ environment. Exposure to the N-protein then activates the immune system to trigger antibody production to destroy infected cells where N-protein or its fragments are displayed on the cell surface. This way, anti-N antibodies could prevent the further spread and infection of the virus. In fact, back to 1986, anti-N antibodies against mouse hepatitis viruses (MHVs), members of the coronavirus family, were reported to protect mice from hepatitis virus type 3 (MHV-3)-induced acute disease, and anti-N antibodies also protected mice against a challenge of lethal mouse hepatitis viruses type 2 (MHV-2) [8,9]. Several reports claimed N-protein as vaccine candidate for SARS-CoV [10]. In 2020, studies have been reported by Ahlen et al. and Dutta et al. which support the notion that N-protein is a good target for SARS-CoV-2 vaccine development [11,12]. In this study, we provide more experimental data from animal models to support this proposal.

## Materials and methods

### Nucleocapsid (N) protein sequences alignments

Amino acid sequence of Severe Acute Respiratory Syndrome Coronavirus type 2 (SARS-CoV-2), severe acute respiratory syndrome coronavirus (SARS-CoV), BAT Coronavirus (BAT-CoV) and BAT Severe Acute Respiratory virus (BAT SARS) were obtained from Uniprot.org. The amino acid sequence of 4 N-proteins were aligned by using Claster W of DNASTAR and GeneDoc software were used to analyze.

### Preparation of GST-N-protein

GST-N-protein bacterial clone was kindly supplied by Dr Yee Joo TAN (Monoclonal Antibody Unit, IMCB, A*STAR, Singapore). In brief, the clone was cultured overnight in small scale (5 ml) Luria-Bertani (LB)/ ampicillin media was transferred to large scale (500 ml) LB/ ampicillin and let grow until required amount was obtained as measured by optical density (OD). The media were added with isopropyl β-D-1-thiogalactopyranoside (IPTG) for induction of protein expression and incubated overnight at room temperature followed by centrifuged at 5,000 rpm for 10 min. The pellet was then incubated with GST extraction buffer on ice for 15 min and sonicated for 5 min. The lysate was centrifuged, and the supernatant was filtered using 0.45 µM filter. The filtrate was added into purification column, after washing with PBS and packed with 1 ml of 50% glutathione resin, for 1 h at 4°C. The column was drained, washed with GST buffer and eluted with elusion buffer to collect GST-N protein [13].

### Animal

Animal experiments were performed in Biological Resource Center (BRC), Agency for Science Technology and Research (A*STAR) under the approval of Institutional Animal Care and Use Committee (IACUC), A*STAR with the approval number #IACUC 191441. Eight-week-old female BALB/c mice (In vivos, Singapore) and FVB mice (Biological Resource Center, A*STAR, Singapore) were used. Animals were killed by cervical dislocation after fully anaesthetised under ketamine/xylazine (150 mg/10 mg per kg) anaesthesia.

### Vaccine preparation and injection

GST-Nucleocapsid protein (GST-N- protein) was purified from GST-N-protein bacterial clone. Nucleocapsid peptides; Peptide #1, Peptide #2 and Peptide #3 were synthesized by Genemed Synthesis, Inc (U.S.A.). GST-N-protein (75 µg) or Peptide #3 (20 µg) in 100 µl of PBS were mixed thoroughly with 100 µl of complete Freund’s adjuvant for first vaccination and incomplete Freund’s adjuvant for booster vaccination (Thermo Scientific). The mice were immunized by intraperitoneal injection of each vaccine in 2-week intervals for 3 or 4 times. Adjuvant only was injected in a group of mice as negative control. Blood samples in Eppendorf tube, and serum was prepared. The antibody titer was measured by ELISA.

### Preparation of serum samples for ELISA

Collected blood at different time point were centrifuged at 5000 rpm for 15 min and serum was obtained by collecting supernatant. Collected serum was stored at -80°C. 2-fold serial dilution of serum was done in PBS starting from the ratio of 1:512 which is the same as 2-fold per step in 9 steps (Table 1).

**Table 1:** Steps of 2-fold serial dilution of mouse serum

Dilution steps	Double dilution (µl)	Total volume after each step
1	1+1	2
2	2+2	4
3	4+4	8
4	8+8	16
5	16+16	32
6	32+32	64
7	64+64	128
8	128+128	256
9	256+256	512
1 µl of serum was mixed with 512 µl of buffer for dilution step 9		
serial 2 times dilution was done from step 10 onwards		

### Classified specific antibody subtypes induced in mice vaccinated with whole N-Protein or Peptide#3

96-well plates (IWAKI, Japan) were coated with 100 µl of GST-N-protein (0.5 µg/ml) or peptides (0.2 µg/ml) and incubated at 4°C overnight. The coated plates were then blocked with blocking buffer, 3% bovine serum albumin (BSA) at room temperature (RT) for 1 h and washed with PBS-Tween (PBS with 0.05% Tween-20). 0.1 ml of diluted mouse serum (2-fold serial dilution) was added to each well of the plates and incubated at 37°C for 1.5 h followed by washing. Different subtypes of bound antibody were detected by incubation with different horseradish peroxidase (HRP)–conjugated antibodies at 37°C for 1 h. Anti N-Protein IgM antibody was detected by goat anti-mouse IgM-HRP Antibody (Invitrogen 626820). Subtype of anti N-Protein IgG2a and IgG1, were detected by goat anti-mouse IgG2a-HRP (Invitrogen A10685) and goat anti-mouse IgG1-HRP (Invitrogen A10551) secondary antibodies. Anti N-protein IgG (whole IgG) were detected by goat anti-mouse IgG-HRP (H+L) secondary antibody (Invitrogen 31430). The plates were washed with PBS-Tween subsequently and incubate with 100 µl of tetramethylbenzidine (TMB) peroxidase substrate (Thermo Scientific). The HRP and TMP reaction was stopped by 100 µl of 2 M H_2_SO_4_. Optical density (OD) of the reaction was read using a plate reader (Tecan). Normal mouse serum at 9 steps dilution was used as control. OD > 3 times of normal mouse serum was considered as positive signal. The positive signal at specific steps of dilution was considered as the titer of that sample [13].

### Binding affinity of polyclonal sera to N protein

96-well plates (IWAKI, Japan) were coated with peptides (#1, #2, #3) and N-protein at different concentration, 5 or 20 ng per well at 4°C overnight, blocked by 3% BSA at RT for 1 h and washed with PBS-Tween followed by incubation using the serum samples (1:1000 and 1:2000 dilution) for 1.5 h. The plates were washed with PBS-Tween and incubated with goat anti-mouse IgG-HRP (H+L) secondary antibody (Invitrogen 31430) for 1 h, then washed again with PBS-Tween, incubated with TMB buffer and read OD as described above.

### Immuno-profiling of blood from unvaccinated and vaccinated mice

Whole blood from mice was lysed to remove RBCs with ACK Lysing Buffer (Gibco, A1049201) for 10 min at RT. The remaining single-cell suspensions were then incubated with Zombie UV Fixable Viability dye (BioLegend) for 30 min at 4°C, approximately 300,000–500,000 cells were used per stain. Incubation with anti-CD16/32 (clone 2.4G2; BD Biosciences) for 30 min at 4°C was done to block non-specific labelling before multiplex labelling for 30 min at 4°C with the following antibodies from BioLegend: Brilliant Violet 711 anti-mouse CD3e (clone 145-2C11), PE-Cy7 anti-mouse CD4 (clone RM4-5), Brilliant Violet 786 anti-mouse CD8a (clone 53-6.7), PE/Dazzle 594 anti-mouse CD11b (clone M1/70), APC-Cy7 anti-mouse CD19 (clone 6D5), Brilliant Violet 510™ anti-mouse CD25 (clone PC61), AF488 anti-mouse CD45 (clone 30-F11), APC anti-mouse CD69 (clone H1.2F3) PE/Dazzle 594 anti-mouse CD127 (clone A7R34), Brilliant Violet 421 anti-mouse CD335 (clone 29A1.4), Brilliant Violet 421 anti-mouse IgG (clone Poly4053). And the following antibodies from BD Biosciences: BV711 Anti-Mouse CD3e (clone 145-2C11), APC-Cy7 Rat Anti-Mouse CD19 (clone 1D3), PE Anti-Mouse CD44 (clone IM7), FITC Anti-Mouse CD45 (clone 30-F11), BV650 Anti-Mouse CD62L (clone MEL-14), PE-CF594 Anti-Mouse CD80 (clone 16-10A1), BV786 Anti-Mouse CD138 (clone 281-2), BV510 Anti-Mouse CD273 (clone TY25), APC Anti-Mouse IgD (clone 11-26c.2a). All samples were run on a BD LSR II flow cytometer (BD Biosciences) and analyzed using the FlowJo software 10.5.3 (FlowJo).

### Cytokine array

Mouse serum cytokines from unvaccinated and vaccinated mice were analyzed with the RayBiotech mouse cytokine array C1 (Cat: #AMM-CYT-1-8) using the provided experimental protocol unless otherwise indicated. In brief, blots were blocked with 2 ml of provided blocking buffer and incubated at RT for 30 min. About 6 µl of serum in each sample was diluted with blocking buffer to a total 500 µl volume. Arrays were incubated with dilute serum sample at 4°C for overnight followed by washing with provided washing buffers according to the standard protocol. Next arrays were incubated with 500 µl of pre-diluted biotinylated antibody cocktail at RT for 4 h. Arrays were then washed again with provided washing buffers according to the standard protocol, incubated with 500 µl of ×1 HRP-Streptavidin at RT. Chemiluminescence detection was done after washing and incubating with detection buffers.

### Generation of mouse monoclonal antibody (6H3) against N-protein

SARS-CoV N-protein hybridoma clone (6H3) was generated by fusion of splenocytes from SARS-CoV N-protein immunized BALB/c mice and SP2/0 myeloma cells. Hybridoma clone were cultured in DMEM medium. A total of 5 × 10^5^ of cells were inoculated into the peritoneal cavity of female BALB/c mice. The released antibody from hybridoma cells was accumulated gradually as ascites fluid. The ascites was collected by abdominal paracentesis after the mice gained 20% of body weight. The ascites fluid was centrifuged at 200 ×*** g*** for 10 min and the supernatant was stored at −80°C [13].

## Results

### N-protein of SARS-CoV-2 was conserved with other coronaviruses

**Figure 1:**
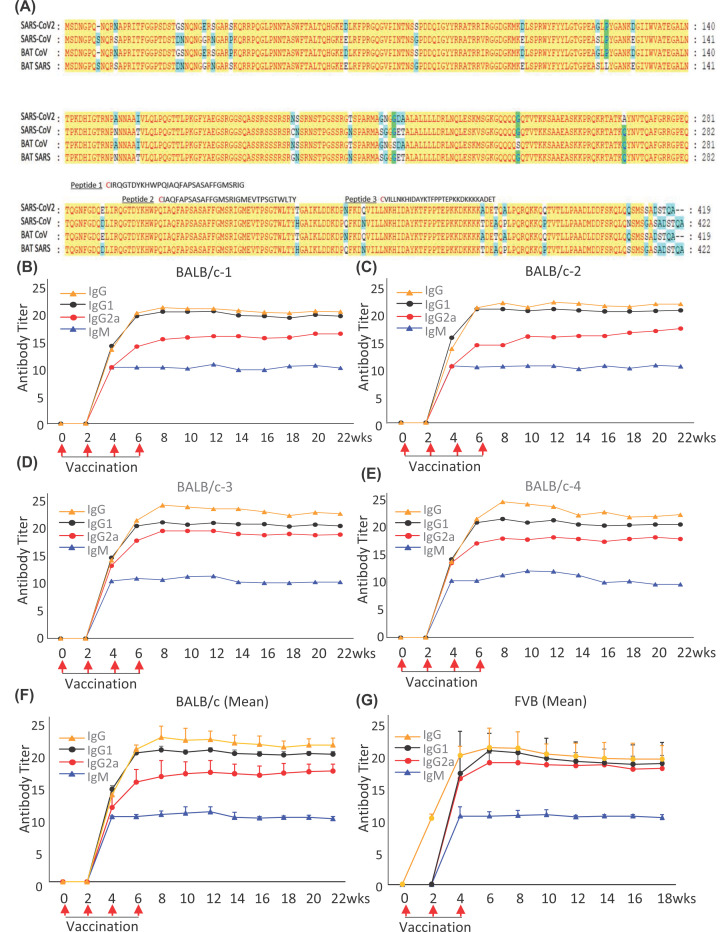
N-protein is an excellent immunogen for activating antibody response (**A**) N-protein alignment from 4 different Coronaviruses (CoVs): 1. Severe acute respiratory syndrome coronavirus type 2 (SARS-CoV-2); 2. Severe acute respiratory syndrome coronavirus (SARS-CoV); 3. BAT severe acute respiratory (BAT SARS); 4. BAT coronavirus (BAT-CoV). Yellow color indicates the similarity of sequences among four viruses. The location of Peptide #1, #2, #3 (30 amino acid sequences) is also mentioned. (**B–E**) The whole N-protein vaccination was repeated four times (2-week interval). Red arrow indicates the time points for vaccination. Blood samples were taken before vaccination followed by every 2 weeks until 22nd week. Serum antibodies were detected by using anti-IgM, -IgG1, -IgG2 and anti IgG (H+L) horseradish peroxidase (HRP) conjugated antibodies. Antibody responses can be detected after 2nd vaccination and sustained till last sample collection in mouse#1 (B), #2 (C), #3 (D) and #4 (E). (**F**) Mean data of antibody productions in the BALB/c mice (*n*=4). (**G**) Mean antibody production of N-protein vaccination in FVB mice (*n*=3). Data represent mean ± S.D.

Coronaviruses are characteristically named for the crown-like spikes on the surface. There are seven coronaviruses transmittable to humans. We have aligned the amino acid sequences of nucleocapsid proteins from 4 different coronaviruses (CoVs) which can give rise to severe acute respiratory syndrome in humans. The current pandemic severe acute respiratory syndrome coronavirus type 2 (SARS-CoV-2), 2003 Pandemic severe acute respiratory syndrome coronavirus (SARS-CoV), BAT coronavirus (BAT-CoV) and BAT severe acute respiratory virus (BAT-SARS) were aligned (Figure 1A), and there is 90% similarity of amino acid sequences. Conserved domains were highlighted in yellow.

### N-protein immunization can produce high and sustainable anti-N-protein antibody subtypes

Immunization using N-protein was performed in BALB/c mice and the antibody response at different time intervals was analyzed. Serum level of anti-N IgM, IgG2a, IgG1 and IgG were measured by ELISA method to evaluate the immune response profile. IgG1 indicates the humoral immune response and IgG2a indicates the cellular immune response. After second vaccination, IgM, IgG2a, IgG1 and IgG antibodies reached to a detectable range. The antibody subtypes were highest after fourth vaccination and stayed at the plateau phase throughout the time points beyond 5 months in each BALB/c mouse (Figure 1B–E). Vaccinations significantly boosted the concentrations of all antibody subtypes, IgM (*P*<0.001), IgG2a (*P*<0.001), IgG1 (*P*<0.0001), and IgG (*P*<0.0001, one-way ANOVA). In the comparison between 2 weeks (2 weeks after first vaccination) and 4 weeks (2 weeks after second vaccination) onwards, it was observed that the increase in antibody concentration of IgG1 was higher than IgG2. Figure 1F showed mean value of Figure 1B–E. Data represented mean ± S.D, *n*=4.

To confirm the above N-protein vaccination results, we then performed the same N-protein immunization in another mouse strain (FVB mice). The same trend of antibody production was also detected in FVB mice (Figure 1G). By ELISA assays, we showed that mice vaccinated with whole N-protein produced a high titer of specific anti-N antibody subtypes: IgM, IgG, IgG1, IgG2a (*P*<0.0001, one-way ANOVA), suggesting that N-protein is an excellent antigen with high immunogenicity to evoke an immune response and the production of anti-N specific antibodies at high titers. Data represented mean ± S.D, *n*=3.

### Peptide #3 derived from N-protein conserved domain could be a good immunogen

**Figure 2:**
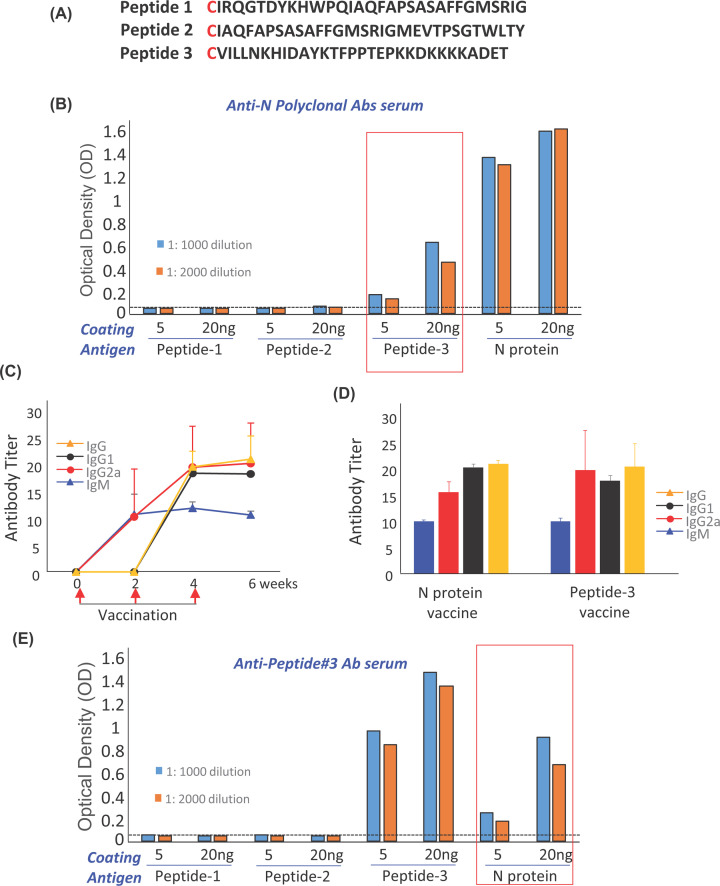
Peptide #3 is a good immunogen (**A**) Peptide sequences were selected based on N-protein conserved regions highlighted in yellow (Figure 1A). (**B**) Binding of anti N-polyclonal Abs serum and peptides were tested by ELISA. Whole N-protein was used as control. Peptides, #1, #2, #3 and N-protein were coated respectively with 5 ng and 20 ng/well. Anti-N polyclonal Abs serum (from Figure 1A mouse at 1:1000 and 1:2000 dilutions) was incubated in each well followed by washing, and incubation with secondary anti-mouse IgG (H+L) (HRP). The optical density (OD) was measured. Anti-N polyclonal Abs bind not only whole N-protein but also enriched binding to Peptide #3, the highest OD compared with Peptide #1 and #2. (**C**) BALB/c mice were immunized with Peptide#3 in 2-week interval, 3 repeats. Red arrow indicates each vaccine time point. Antibody subtype in *Anti-Peptide #3 Ab serum* were classified by using anti-IgM, -IgG1, -IgG2a and -IgG (H+L) horseradish peroxidase (HRP) conjugated antibodies. Data represents mean ± S. D, *n*=3. (**D**) Comparison osf antibody subtype induction in N-protein vaccine and Peptide #3 vaccine after vaccination for three times. (**E**) *Anti-Peptide #3 Ab serum* (from C) were tested by Elisa for the binding capacity. *Anti-Peptide #3 Ab serum* binds to Peptide #3 and whole N-protein, detected by anti-mouse IgG (H+L) (HRP).

N-protein vaccination produces polyclonal antibodies against epitopes throughout the whole N-protein sequences (419 amino acids). To screen immunogenicity domains in the N-protein, we selected and synthesized peptide #1- #3, each was 30 amino acids in length (Figure 2A) along the N-protein conserved regions (Figure 1A). We examined the three peptides for the binding capacity of the anti-N polyclonal antibodies by ELISA assays. Coating 5 ng or 20 ng per well for each peptide side by side with whole N-protein, incubated with serum sample taken after the fourth immunization, at 1:1000 and 1:2000 dilutions. Peptide #3 showed the best binding activity (Figure 2B, red box indicated) compared to peptide #1 and #2. The OD of peptide #1 and Peptide #2 were nearly 10 times lower than peptide #3. The OD of Peptide #3 was nearly half of N-protein OD, suggesting Peptide#3 could be a good immunogen and potentially be combined with the cocktail of conventional influenza vaccines as a general safe vaccine against influenza as well as SARS-CoV.

### Peptide #3 vaccination could induce high and sustainable antibody production similar to N-protein vaccine

Peptide #3 mixed with Freund adjuvant was immunized to BALB/c mice, 3 times in a 2-week interval to confirm that peptide #3 alone could be a good immunogen for vaccination to evoke host immune system to produce antibody specific to N-proteins of CoVs. Similar to N-protein vaccination, serum IgM, IgG1, IgG2a and IgG antibodies were detected by ELISA after the second vaccine dose. The pattern and quantity of rise in antibody titer is similar to whole N-protein vaccine (Figure 2C–E). To test the binding of Peptide #3 polyclonal sera to N-protein, peptide #1 and #2, ELISA assay was done using the serum samples (1:1000 and 1:2000 dilution) taken 2-week after the third Peptide #3 vaccination. As expected, Peptide #3 polyclonal sera showed no bindings to peptide #1 and #2 (as negative controls), binding best to its own, and good affinity to whole N-protein (Figure 2E).

### N-protein vaccination results in the accumulation of memory T cells

To investigate if N-protein vaccination can lead to the memory immune cell accumulation, vaccination of 8-week-old BALB/c mice were carried out once every two weeks with a combination of Freund’s adjuvant and N-protein for four times. Vaccinated mice were then bled and killed 16 weeks after the last vaccination to determine if they had elevated levels of memory cells compared with unvaccinated mice.

**Figure 3:**
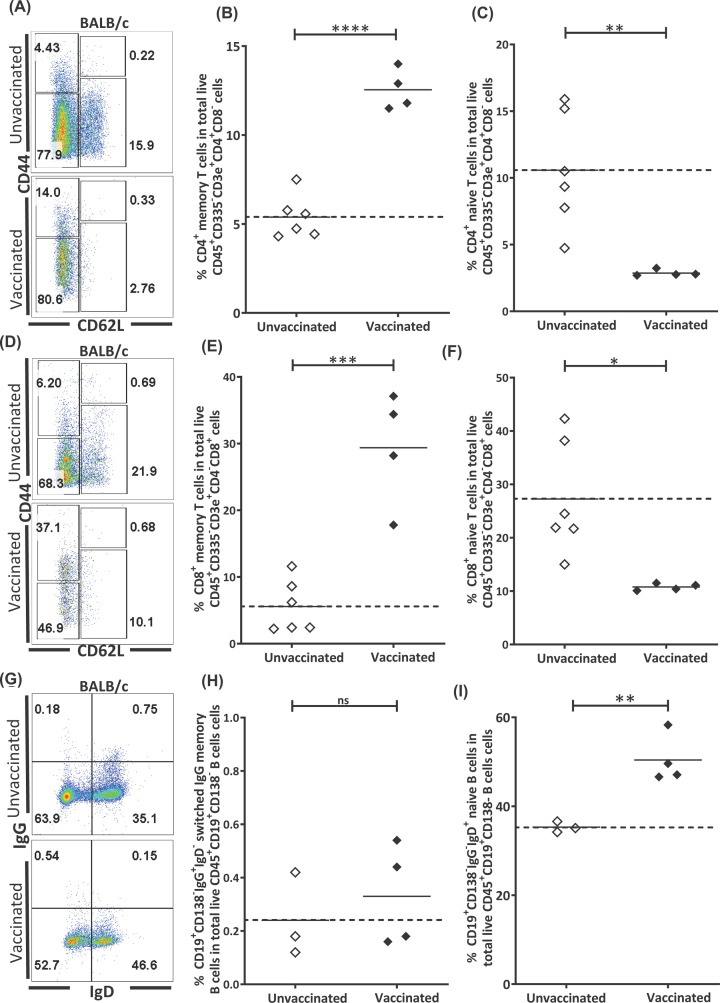
Vaccination results in an increased frequency of CD4^+^ and CD8^+^ memory T cells and a decreased frequency of memory T cells (**A**) Representative CD62L and CD44 staining on live CD45^+^CD3^+^CD335^−^CD4^+^CD8^−^ T cells from the blood of BALB/c mice. Mice were either unvaccinated (upper panel) or vaccinated with N-protein (vaccinated mice, lower panel). (**B**) Change in the percentage of live CD44^+^CD62L^−^ memory T cells as a proportion of total live CD45^+^CD3^+^CD335^−^CD4^+^CD8^−^ T cells in unvaccinated and vaccinated mice. (**C**) Change in the percentage of live CD44^−^CD62L^+^ naïve T cells as a proportion of total live CD45^+^CD3^+^CD335^−^CD4^+^CD8^−^ T cells in unvaccinated and vaccinated mice. (**D**) Representative CD62L and CD44 staining on live CD45^+^CD3^+^CD335^−^CD4^−^CD8^+^ T cells from the blood of BALB/c mice. Upper panel represents unvaccinated and lower panel represents unvaccinated mice. (**E**) Change in the percentage of live CD44^+^CD62L^−^ memory T cells as a proportion of total live CD45^+^CD3^+^CD335^−^CD4^−^CD8^+^ T cells in unvaccinated and vaccinated mice. (**F**) Change in the percentage of live CD44^−^CD62L^+^ naïve T cells as a proportion of total live CD45^+^CD3^+^CD335^−^CD4^−^CD8^+^ T cells in unvaccinated and vaccinated mice. (**G**) Representative IgD and IgG staining on live CD45^+^CD19^+^CD138^−^ B cells from the blood of unvaccinated (upper panel) and vaccinated (lower panel) Balb/c mice. Change in the percentage of (**H**) naïve IgD^+^ B cells and (**I**) IgG^+^ class-switched memory B cells. Data representing mean ± SEM, *n*=4. *P≤0.05, **P≤0.01, ***P≤0.001, ****P≤0.0001.

Because former SARS (SARS-CoV) patients possess long-lasting memory T cells against N-protein which are also cross-reactive to the N-protein of SARS-CoV-2 [14], we analyzed the memory T-cell compartment of our mice. Circulating live CD45^+^CD3^+^CD335^−^CD4^+^CD8^−^CD44^+^CD62L^−^ (Figure 3A,B) and CD45^+^CD3^+^CD335^−^CD4^−^CD8^+^CD44^+^CD62L^−^ (Figure 3D,E) memory T cell frequencies were significantly (CD4^+^, *P*=0.0000129; CD8^+^, *P*=0.000306, *t*-test) increased in vaccinated mice compared with unvaccinated mice, suggesting that our vaccination protocol can successfully induce a robust and lasting memory CD4^+^ and CD8^+^ T-cell population.

Additional phenotypic analysis of T-cell subpopulations reveal a corresponding decrease in the proportion of circulating live CD45^+^CD3^+^CD335^−^CD4^+^CD8^−^CD44^−^CD62L^+^ (Figure 3A,C) and CD45^+^CD3^+^CD335^−^CD4^−^CD8^+^CD44^−^CD62L^+^ (Figure 3D,F) naïve T cell levels (CD4^+^, *P*=0.00817; CD8^+^ *P*=0.0160, *t*-test) in vaccinated mice compared with unvaccinated mice, supporting our observation that our vaccination protocol results in a decrease in antigen naïve T cells and an elevated frequency of antigen experienced memory CD4^+^ and CD8^+^ T cells.

In contrast, the change in live CD45^+^CD19^+^CD138^−^IgD^+^IgG^−^ naïve B cells and CD45^+^CD19^+^CD138^−^IgD^−^IgG^+^ class-switched memory B cells is less distinct. The frequency of both IgG class-switched memory B cells in vaccinated mice is similar to their unvaccinated counterparts, while the frequency of naïve B cells is elevated in vaccinated BALB/c mice (Figure 3G–I). The lack of a permanent large increase in the frequency of memory B cells in our vaccinated mice may indicate that T cells may be more important for mediating long term immune response against SARS-CoV-2 and may explain why some former SARS-CoV-2 patients experience a decline in antibodies several months after recovery.

### Vacination of N-protein with complete Freund’s adjuvant (CFA) can induce the secretion of pro-inflammatory memory cell and T_H_1 associated cytokines

**Figure 4:**
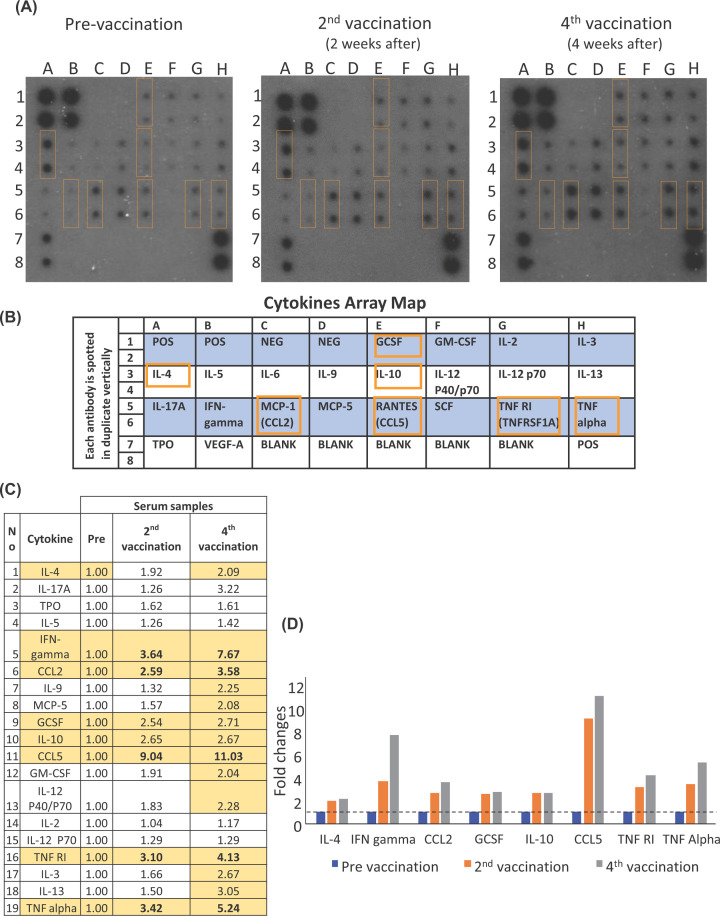
Vaccination of whole N-protein with complete Freund’s adjuvant (CFA) can induce the secretion of pro-inflammatory memory cell and T_H_1 associated cytokines Cytokine arrays were performed for pre-vaccination, second vaccination (2 weeks after second vaccination sample) and fourth vaccination (4 weeks after fourth vaccination sample) mice sera. (**A**) Cytokine array blot of pre- (first blot) and post-vaccination mouse sera (second blot for 2 weeks after second vaccination and third blot for 4 weeks after fourth vaccination). (A to H) indicate columns and 1 to 8 indicate rows of the blots. The orange box indicates the cytokines which increased more than 2 folds than pre-vaccination sample. (**B**) Map of cytokine array used in each blot of (A). (**C**) The table indicating fold increases in cytokine level based on pre-vaccination sample. The cytokines which increased more than 2 folds after fourth vaccination were highlighted with yellow. (**D**) Bar graph of cytokines from (C) which were more than 2-fold increase compared to pre-vaccination sample.

Cytokine array studies of mouse serum suggest that our vaccinated mice have elevated levels of pro-inflammatory cytokines and chemokines such as CCL2, CCL5, IFN-γ, TNF-α, TNF-RI, GCSF, IL-4, and IL-10 compared to unvaccinated mice (Figure 4A–D). A subsequent cycle of vaccination (fourth vaccination) also results in an increased level of these cytokines compared with a prior cycle (second vaccination), suggesting that repeated vaccinations with N-protein can result in progressively elevated cytokine levels in mice (Figure 4C,D) and likely enhanced immune responses during subsequent vaccinations due to the accumulation of memory immune cells.

### Generation of anti N-protein antibody against nucleocapsid N-protein for future therapeutics

**Figure 5:**
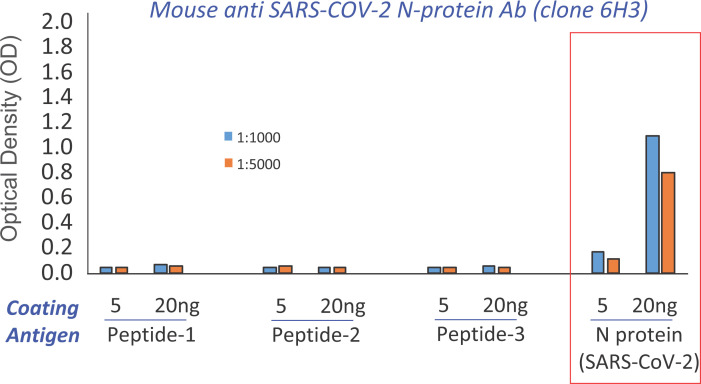
Clone 6H3 mouse monoclonal antibody binds to SARS-CoV-2 N-protein with good affinity ELISA was done to analyze the binding affinity of peptides and N-protein (SARS-CoV2) to in house produced mouse anti *SARS-CoV Ab (clone 6H3)*. ELISA plate was coated with 5 ng and 20 ng/ well of different peptides and N-protein (SARS-CoV2). Mouse 6H3 antibodies were diluted at 1:1000 and 1:5000. The binding of antibody was detected by anti-mouse IgG (H+L) (HRP). The optical density (OD) was measured.

In 2003, we generated a monoclonal antibody (clone 6H3) against SARS-CoV, Anti-SARS-CoV-N-protein antibody clone (6H3). Since SARS-CoV and SARS-CoV-2 shared >90% similarity in its N-protein amino sequence (Figure 1A), there is a possibility of SARS-CoV antibody binding to SARS-CoV-2 N-protein with good affinity. We demonstrated Anti-SARS-CoV-N-protein antibody clone (6H3) cross-reacting with SARS-CoV-2 N-protein, using ELISA assay to access the binding affinity. ELISA plate was coated with 5 and 20 ng/well of Peptide #1, #2, #3 and N-protein (SARS-CoV-2). Each well was incubated with Anti-SARS-CoV-N-protein antibody clone (6H3) diluted at 1:1000 and 1:5000, followed by incubation with secondary goat anti-mouse IgG-HRP antibody. The measurement of optical density showed SARS-CoV-N-protein antibody could not bind to Peptide #1, #2, and #3 but bind strongly to SARS-CoV-2 N-protein depending upon concentration (Figure 5), suggesting clone 6H3 epitope presents in the whole N-protein but not in these three peptides. The mouse antibody (6H3) is currently developing as the first-in-class humanized antibody in future to treat patients infected with SARS-CoVs virus.

## Discussion

It is known that the SARS coronavirus (SARS-CoV and, SARS-CoV-2) infects target cells via S-protein receptor binding domain (RBD) and ACE2 receptor interactions [15,16]. Thus, the SARS-CoV-2 S-protein and its RBD were then selected as the leading target antigens in recent vaccine development [3,17,18], expecting the generation of effective neutralizing antibodies to block SARS-CoV-2 viral entry.

In theory, the *Spike protein* should be considered for vaccine developments. However, the heavy glycosylation and mutations of the S-protein have posed challenges for this ideal proposal due to the infeasibility of large productions of S-protein in vitro to mimic viral natural antigens. As a result, the *Spike mRNAs,* currently serving as a lead vaccine against SARS-CoV-2, has caused several uncertainties raised for this new mRNAs vaccine strategy: first, how effective is the translation and post-translational modification of *Spike mRNAs* *in vivo* to mimic actual viral Spike protein and to acutely guide the host immune system in producing anti-Spike specific antibodies? Second, how long does such vaccine immunity (if any) last? Third, how can the current *Spike mRNAs* vaccine strategy overcome 'viral immune escape’ since multiple mutations in S-proteins may enable the mutated virus strains to escape from immunity? Therefore, there is an urgency to stay ahead of the pace of viral mutation and spread with new strategies.

Wang et al. reported that volunteers injected with either the Moderna (mRNA-1273) or Pfizer–BioNTech (BNT162b2), S-protein mRNA vaccine against SARS-CoV-2 demonstrated high titers of IgM and IgG antibodies against SARS-CoV-2 S protein and RBD [19]. It is also reported that the plasma neutralizing activity and relative numbers of RBD-specific memory B cells of vaccinated individuals are similar to patients who recovered from natural infection [19–21]. The presence of neutralizing antibodies against SARS-CoV-2 S-protein and its RBD does not confer complete protection against SARS-CoV-2 infection in vaccinated individuals, even in a subset of recently vaccinated individuals, still contract SARS-CoV-2 [22,23].

In addition, it has been reported that the titers of SARS-CoV-2 neutralizing antibodies decline fairly and rapidly, with some individuals reporting close to baseline neutralizing antibody levels as soon as 2 months post-infection [24–26]. The SARS–CoV-2 S-protein also has a relatively lower amino acid similarity (76%) compared to the SARS–CoV S-protein with a higher rate of mutation compared to the more conserved [90] % N gene [27–31]. These data suggest that while anti-S protein antibodies may be key for controlling viral titers during an ongoing infection, other immune mediators may be responsible for conferring long-term immunity to SARS-CoV-2. In particular, N-protein, an ‘Intra-viral’ protein, may be underappreciated and will be investigated in the present study.

The N-protein is highly immunogenic and is the most abundant viral protein during coronavirus infections [2,32]. It is also a major target for antibody and T-cell responses [33]. Importantly, non-neutralizing antibodies against N-protein can protect mice against some other viruses, such as the mouse hepatitis virus [8,9] and influenza A virus [34]. Recently, it has also been reported that vaccine targeting N-Protein prevents pneumonia in Rhesus Macaques SARS-CoV-2 infection model [35]. N-protein is commonly externalized on the cell surface membrane of infected cells, and can act as a potential target for both antibody and T-cell responses [36]. The memory T cells may play a critical role in conferring long-term immunity to SARS-CoV-2. Grifoni et al. reports the induction of robust CD4^+^ and CD8^+^ T cells in convalescent SARS-CoV-2 patients. Surprisingly even some non-exposed individuals demonstrate T-cell reactivity against SARS-CoV-2 epitopes, suggesting that prior infections in these individuals could also enhance immunity against SARS-CoV-2 [36]. Bert et al. also demonstrates that former SARS-CoV patients possess long lasting memory T cells which are reactive to N-protein over 17 years after the SARS epidemic in 2003 [14]. These memory T cells were also highly cross-reactive to the SARS-CoV-2 N-protein, suggesting that these individuals may be less susceptible to SARS-CoV-2 infection and other similar coronavirus [14]. Other animal model studies involving vaccination with SARS-CoV N-protein have also demonstrated robust SARS-specific T-cell proliferation and cytotoxic responses [37,38]. N-protein specific CD8^+^ T cells also protect against infectious bronchitis virus model in chickens. These data suggest that T cells are essential for mediating long-term immunity.

In short, our findings have shown that N-protein or its peptide vaccinations provide reasonably long immunity. Moreover, vaccinated mice remained active and with steadily increasing body weights during the course of the experiment, similar to that of the control group and did not detect any safety issue. Importantly, N-protein is strongly recognized by the host immune system as there is a consistent presence of anti N-protein antibodies in the sera of SARS-CoV2 infected patients [39]. On the potential of anti N-protein antibodies in the prevention of infection, dominant helper T-cell epitopes in the N-protein of SARS-CoV have been identified to assist in antiviral neutralizing antibody production [40]. The anti N-protein antibodies have been previously shown to confer protection against several types of lethal influenza A viruses [41–43]. A combination of neutralizing antibodies targeting S protein and its RBD, anti N-protein antibodies, and memory T cells against N-protein epitopes may be essential to confer long-term protection against SARS-CoV-2.

We identified and reported a peptide gave similar results as that of the whole N-protein, suggesting this peptide is not only the major epitopes in the N-protein but also sufficient to elicit immunity in the host.

Immunological memory depends on the creation of memory T and B cells as part of a primary immune response [44]. Memory cells often respond more rapidly to antigen resulting in faster production of cytokines and expansion to form effector cells [45,46]. CCL5 is a pro-inflammatory chemokine essential for T-cell activation and for directing the migration of immune cells such as T cells, monocytes, NK cells, and dendritic cells to sites of infection. Memory T cells constitutively express high levels of CCL5 mRNA compared with their naïve T cell counterparts and T-cell receptor (TCR) stimulation alone was sufficient to induce the production of CCL5 in memory T cells [47]. The elevated levels of memory CD4^+^ and CD8^+^ T cells was observed post stimulation, coupled with an enhanced serum CCL5 levels in subsequent vaccinations indicates that the pro-inflammatory memory T-cell response is enhanced by multiple vaccination cycles.

IFN-γ is a pro-inflammatory cytokine produced by innate immune cells such as natural killer (NK) cells and innate lymphoid cells, and adaptive immune cells such as T_H_1 cells. While IFN-γ is produced by innate cells early in the immune response, a high and sustained level of IFN-γ production usually requires the TCR mediated detection of specific antigens (e.g. N-protein) by T_H_1 cells [48]. The elevated production of IFN-γ observed (Figure 4A–D) after multiple vaccinations may indicate that vaccination with CFA and N-protein induces a T_H_1 mediated immune response. This observation is consistent with prior studies, as CFA induces a strong T_H_1 immune response due to its mycobacterial components [49–51].

Clinically, patients infected by SARS-CoV and SARS-CoV-2 first exhibited antibodies against N-protein, which are the most sensitive for serologic diagnosis [52–56]. Hence, we urge immediate attention both on the evaluation of N-protein as a novel vaccination and on generation of anti-N humanized antibody drugs against Coronaviruses (CoVs). Our data also showed that in-house developed anti N-protein antibody against N-protein of SARS-CoV can recognize/bind to the SARS-CoV-2 N-protein with good affinity. From this, it is proposed that a humanized anti N-protein antibody could potentially be used as a therapy in the eradication of SARS-CoVs infected host cells. In 2012, we had proposed similar strategies of targeting ‘Intra-viral’ proteins as unconventional immunotherapies against viral infections. We suggested one could contemplate to target hepatitis B virus (HBV) X- protein which is localized in the nucleus of infected cells [57,58]. As HBV infection is associated with Hepatocellular carcinoma, targeting X-protein using antibodies will be a therapeutic option for treatment of HCC by destroying infected cells and leaving normal cells unharmed. We are awaiting a New Era of Vaccine prevention and Immunotherapy to treat human diseases.

## Conclusion

To overcome the viral immune escape and the recurrence of SARS-CoVs infections due to the mutation of ‘Extra-viral’ S protein, herein, we join the Vaccines arena by proposing a novel concept of targeting the ‘Intra-viral’ conserved N protein/peptides which have been shown in this study to be excellent immunogens. It is envisioned that N protein/peptides could be combined as a cocktail with conventional influenza vaccines to confer protection against various coronaviruses. Furthermore, we anticipate that the first-in-class humanized antibodies against N-protein could be effective therapeutics for patients infected by coronaviruses.

## Data Availability

All data generated or analyzed during this study are included in this article.

## Abbreviations

BAT-CoV: BAT coronavirus
BAT SARS: BAT severe acute respiratory syndrome
COVID-19: coronavirus disease in late 2019
CoVs: coronaviruses
ELISA assay: enzyme-linked immunosorbent assay
GST: Glutathione S-transferase
IPTG: isopropyl β-D-1-thiogalactopyranoside
LB media: Luria–Bertani media
MERS: Middle East respiratory syndrome
MERS-CoV: Middle East respiratory syndrome coronavirus
N-protein: nucleocapsid protein
OD: optical density
PBS: phosphate-buffered saline
SARS: severe acute respiratory syndrome
SARS-CoV: Severe acute respiratory syndrome coronavirus
SARS-CoV-2: Severe acute respiratory syndrome coronavirus 2
